# Validity of Cognitive Tests for Non-human Animals: Pitfalls and Prospects

**DOI:** 10.3389/fpsyg.2020.01835

**Published:** 2020-08-31

**Authors:** Michèle N. Schubiger, Claudia Fichtel, Judith M. Burkart

**Affiliations:** ^1^Evolutionary Cognition Group, Department of Anthropology, University of Zurich, Zurich, Switzerland; ^2^World Ape Fund, London, United Kingdom; ^3^Behavioural Ecology and Sociobiology Unit, German Primate Center, Göttingen, Germany; ^4^Leibniz ScienceCampus “Primate Cognition”, Göttingen, Germany

**Keywords:** comparative psychology, cognitive testing, test design, task validity, contextual variables, non-cognitive factors, individual differences, primates

## Abstract

Comparative psychology assesses cognitive abilities and capacities of non-human animals and humans. Based on performance differences and similarities in various species in cognitive tests, it is inferred how their minds work and reconstructed how cognition might have evolved. Critically, such species comparisons are only valid and meaningful if the tasks truly capture individual and inter-specific variation in cognitive abilities rather than contextual variables that might affect task performance. Unlike in human test psychology, however, cognitive tasks for non-human primates (and most other animals) have been rarely evaluated regarding their measurement validity. We review recent studies that address how non-cognitive factors affect performance in a set of commonly used cognitive tasks, and if cognitive tests truly measure individual variation in cognitive abilities. We find that individual differences in emotional and motivational factors primarily affect performance via attention. Hence, it is crucial to systematically control for attention during cognitive tasks to obtain valid and reliable results. Aspects of test design, however, can also have a substantial effect on cognitive performance. We conclude that non-cognitive factors are a minor source of measurement error if acknowledged and properly controlled for. It is essential, however, to validate and eventually re-design several primate cognition tasks in order to ascertain that they capture the cognitive abilities they were designed to measure. This will provide a more solid base for future cognitive comparisons within primates but also across a wider range of non-human animal species.

## Introduction

Comparative psychologists design and use cognitive tests to investigate and compare cognitive performance and capacities of extant non-human animal species and humans (mainly children). The ultimate goal is to better understand how animal minds are organized and to reconstruct the evolution of mind, including the human one. To date, a variety of animal species has been compared in cognitive studies, ranging from mammals (terrestrial and aquatic ones) to birds (e.g., Lambert et al., 2018), fish (e.g., Bhsary and Brown, 2014), reptiles (e.g., Wilkinson and Huber, 2012) and invertebrates such as cephalopods (mainly octopuses: Mather and Kuba, 2013; Amodio et al., 2019) or insects (mainly bees: Chittka, 2017; Solvi et al., 2020). The rationale behind selecting certain species for cognitive studies is typically a low or high degree of variation in brain size or socio-ecological factors such as breeding systems, social structures or feeding ecologies, to better understand the selective pressures driving cognitive abilities. Of particular interest for comparative psychologists are cognitive comparisons between larger-brained species (e.g., non-human primates, elephants, dolphins, and birds from the corvid and parrot families; Tomasello and Call, 1997; Pepperberg, 2002; Emery and Clayton, 2004; Plotnik et al., 2006; Byrne et al., 2009; Maestripieri, 2012; Manger, 2013; Güntürkün, 2014) and smaller-brained species (e.g., rodents, pigeons: Scarf et al., 2011; Matzel and Sauce, 2017) in order to examine the cognitive potential of large brains. More recently, an increasing number of mammalian carnivore taxa are also being studied to better understand the cognitive abilities of this large and in many aspects heterogenous order (e.g., domestic and wild dogs, hyeanas, bears, and meerkats: Townsend et al., 2012; Bensky et al., 2013; Holekamp and Benson-Amram, 2017; Dale et al., 2019; for a review see: Vonk and Leete, 2017).

Valid species comparisons pose an immense challenge for comparative psychology, because obviously, applying a physically identical task is not sufficient to warrant fair species comparisons, which led some researchers to argue this may render meaningful species comparisons impossible (e.g., MacPhail, 1987). A central challenge of contemporary comparative psychology is that both the nature of cognitive abilities and their potential evolution need to be inferred and reconstructed from individual performance scores in human-devised cognitive tests. In comparative psychology, such cognitive tasks often address cognitive abilities from broad domains such as physical cognition to deal with the non-social world and social cognition to deal with the social world (Tomasello and Call, 1997). However, when dealing with more cognitively demanding problems, such as completely novel ones, an individual is required to draw on cognitive resources that can be applied across a wide range of problems from diverse domains. In primates, brain size predicts species-difference in such a global domain-general cognitive ability, which is reflected in an IQ-like performance score of a species (*G*) across a set of diverse tasks and this performance score increases from humans’ evolutionary most distant primate relatives (lowest scores in strepsirrhines) to their closest primate relatives (highest scores in haplorrhines and particularly in great apes; Deaner et al., 2006; Reader et al., 2011). At least in humans, such a domain-general intellectual ability is unequivocal at the individual level (*g*) and increasing evidence in non-human animals suggests considerable evolutionary continuity of *g* (as assessed via reasoning ability and behavioral flexibility), at least within the mammalian lineage (mainly rodents and dogs: as reviewed in Burkart et al., 2017a) including some primates (e.g., tamarins: Banerjee et al., 2009; chimpanzees: Hopkins et al., 2014; orangutans: Damerius et al., 2019). However, among primates, the current evidence is still mixed and controversial with a number of studies finding no support for a *g* (e.g., Herrmann et al., 2007; Amici et al., 2012). This could be consistent with Macphail’s null hypothesis that no major domain-general species differences are expected in primates other than humans (MacPhail, 1985), but could also be an artifact of differences in tests and procedures. In order to empirically assess such potential intra-species differences in domain-general cognition, the focus of comparative cognition studies has recently been shifted from using single tasks toward cognitive test batteries (sets of at least five tasks from various cognitive “domains”). Gaining a better understanding of individual differences will help to achieve the ultimate goal of comparative psychology: meaningful comparisons across a wide range of species.

Cognitive testing of non-human primates and other animals poses several issues for comparative psychology, which become particularly apparent when using test batteries. In this paper, we will therefore use established cognitive test batteries to illustrate some of the most prominent issues researchers generally encounter when testing non-human primates but also other animals. Two of these issues are that the outcomes of cognitive studies should be replicable within individuals or single species and that the cognitive tasks should reliably capture variation in the cognitive abilities they were designed to measure.

### Are the Outcomes of Cognitive Studies With Non-human Primates Replicable?

It is conceivable that at least some comparative cognition tasks do not measure the cognitive abilities they were intended to measure. Besides aspects of the test-design including task sensitivity (i.e., the level of task difficulty should be appropriate to detect individual variation in cognitive performance without producing ceiling or floor effects), several non-cognitive biases may affect the outcomes of both original studies and their replications. Human cognitive test batteries are regularly evaluated regarding psychometric criteria such as their validity (e.g., construct validity: whether the cognitive tasks actually do measure the supposed cognitive abilities their human developers attempted to measure), and reliability (e.g., test-retest-reliability: whether repeated administration of the same tasks to the same participants leads to reproducible outcomes). In comparative psychology with non-human primates and other animals, however, such evaluations are currently largely missing. This might be problematic, especially in light of the recent replication crisis in human psychology highlighting that many original research findings, even from studies using established experimental paradigms, cannot be replicated by fellow researchers (Open Science Collaboration, 2012; Pashler and Wagenmaekers, 2012; Earp and Trafimow, 2015). One consequence of this crisis was a lack of confidence in scientific studies including the used research practices. Comparative psychology has so far largely been spared by this crisis of confidence, but it may be even more susceptible than human psychology owing to its lack of replication studies (e.g., see Farrar et al., 2020 for specific challenges in this field).

The few primate studies that assessed the re-test reliability of primate cognition tests include one that used different test sessions within single cognitive tasks of the Tamarin Test Battery (TTB) as a proxy for repeated testing and found a relatively low correlation between performance scores (Banerjee et al., 2009). In an adapted version of the original memory task from the TTB, marmosets were tested with a no-delay session before and after the six different delay conditions (Schubiger et al., 2016). Although they performed in both above chance level, their performance declined from the former to the latter. This suggested that although the marmosets were still able to solve this very simple task, they might not have been fully motivated anymore to make correct choices after having completed the full memory task with five delay sessions. However, in another study, Hopkins et al. (2014) re-tested a subset of their chimpanzee sample with 13 tasks of the Primate Cognition Test Battery (PCTB, Herrmann et al., 2007) 2 years later and found that overall, performance remained relatively stable (besides improvements in four physical and decreased performance in one social PCTB task). In addition, a recent meta-analysis on the repeatability of cognitive performance from 25 species of six animal classes revealed that cognitive performance could be replicated in both temporal repeatability by comparing performance on several exposures of the same task and contextual repeatability by comparing individual performance on different tasks that measure the same putative cognitive ability (Cauchoix et al., 2018). Hence, to assess the validity of cognitive tests it is important to include, if possible, assessments of repeatability.

In comparative psychology, large samples, particularly of non-human primates, are often not available (ManyPrimates et al., 2019a, b), which limits the statistical power of an empirical study (but see Smith and Little, 2018 for advantages of small sample sizes). The next best option to validate obtained test results in such cases are replications in independent samples of the same species. Some findings were replicated within species and were also found in evolutionarily closely related species, which also establishes the external validity of an optimized task (e.g., a memory task that is often part of test batteries to establish *g*: Schubiger et al., 2016, see also Table 1).

**TABLE 1:** Effect of internal (subject-related) and external (test design-related) non-cognitive factors on an individual’s motivation to participate, attend, and use an appropriate response strategy, and on its cognitive performance in commonly used comparative cognition tasks.

Non-cognitive factors	Participation/attention/response motivation	Performance	Cognitive task(s)/skill(s)	Species	Reference(s)
**1. Internal (subject-related) factors**					
**a) Intrinsic factors**					
*Personality-related motivational differences*					
• High levels of trait anxiety	↓	↓	Reversal learning	Long-tailed macaques	Toxopeus et al., 2005
• High openness	↑	↑	Training	Brown capuchins	Morton et al., 2013
• High assertiveness	↓	↓			
• High levels of boldness vs. shyness	↑ bold	↑ bold	Physical PCTB tasks	Chimpanzees and orangutans	Herrmann et al., 2007
	=	=	Social PCTB tasks		
	↑ bold (males)	=	Physical and social PCTB tasks	Olive baboons	Schmitt et al., 2012
	↓ shy (females)	=		Long-tailed macaques	
	=	=	Physical PCTB tasks	Ring-tailed and ruffed lemurs	Fichtel et al., 2020
	↓ Shy	↑ Shy	Social PCTB tasks	ruffed lemurs	
High emotional reactivity	↓ (males)	=	Object permanence	Common marmosets	Schubiger et al., 2015
**b) Social factors**					
*Rearing conditions, housing conditions, and previous contact with humans*					
• Impoverished rearing conditions	↓	↓	Reversal learning (Transfer Index)	Chimpanzees	Davenport et al., 1973
• Enriched rearing conditions	↑	↑	Mirror self-recognition	Chimpanzees Gorillas	Gallup et al., 1971 Patterson and Cohn, 1994; Posada and Colell, 2007
• Enriched vs. standard nursery-reared	↑	↑	Joint attention (30 BSID tasks) and cooperation (1 IBR task)	Infant chimpanzees	Bard et al., 2014
• Mother-reared vs. nursery-reared	( = )	=	13of the 16 PCTB tasks	Chimpanzees	Hopkins et al., 2014
• Social housing and high levels of human care	(↑)	↑	OTB tasks: inhibitory control, reversal learning, problem solving, causal reasoning	Sumatran and Bornean orangutans	Damerius et al., 2017b
• Degree of previous contact with humans (Human Orientation Index, HOI)	↑ High HOI	↑ High HOI	Problem-solving		Damerius et al., 2017a
*Social tolerance and organization*					
• High social tolerance	(↑)	↑	Cooperation	Tonkean macaques and rhesus macaques Bonobos and chimpanzees	Petit et al., 1992 Hare et al., 2007
	(↑)	↑	Causality scale of the PCTB	Chimpanzees	Herrmann et al., 2010
	(↑)	↑	Theory of Mind scale of the PCTB	Bonobos	
	(↑)	↑	Pointing cups (social task 13 of PCTB), inhibitory control	Barbary macaques, long-tailed macaques, rhesus macaques, and Tonkean macaques,	Joly et al., 2017
		=	All other PCTB tasks		
• High levels of allomaternal care	(=)	=	Physical cognition tasks	Many different non-human primate species	Reviewed in Burkart and van Schaik, 2010; Burkart and van Schaik, 2016
	(↑)	↑	Social cognition tasks		
• High degree of fission-fusion	(↑)	↑	Five inhibitory control tasks	Bonobos, chimpanzees, gorillas, orangutans, long-tailed macaques, spider monkeys, and capuchin monkeys	Amici et al., 2008
	(↑)	↑	Spatial memory (similar to task 1 of the physical PCTB scale), Support (similar to task 8 from the physical PCTB scale)		Amici et al., 2010
**c) Demographic factors**					
*Social rank*	=	=	Physical and social PCTB tasks	Olive baboons and long-tailed macaques	Schmitt et al., 2012
				Ring-tailed and ruffed lemurs	Fichtel et al., 2020
*Sex (gender)*	(↑) males	↑ males	Physical PCTB tasks of the Space scale	Chimpanzees and orangutans	Herrmann et al., 2007
	(↑) females	↑ females	Physical PCTB tasks of the Quantity Scale	Human children	
	=	=	Physical and social tasks of the PCTB	Olive baboons and long-tailed macaques	Schmitt et al., 2012
				Chimpanzees	Hopkins et al., 2014
				Barbary macaques, long-tailed macaques, rhesus macaques, and Tonkean macaques	Joly et al., 2017
				Ring-tailed lemurs, ruffed lemurs, and mouse lemurs	Fichtel et al., 2020
	↓ males	=	Object permanence	Common marmosets	Schubiger et al., 2015
*Age*					
• Older subjects		↑	Causality scale of the PCTB	Bonobos and chimpanzees	Herrmann et al., 2010
		↓	Theory of Mind scale of the PCTB		
		↑	Most physical PCTB tasks	Chimpanzees (only females tested)	Lacreuse et al., 2014
		↓	Spatial memory (physical PCTB task); Attentional state and gaze following (social PCTB tasks)		
	(↓)	↓	Inhibitory control	Common marmosets	Gokcekus, 2020
	(=) curious subjects	= curious subjects			
**2. External (test design-related) factors**					
**a) Task format**					
*Physical cognition tasks*					
• Possibility to rake-in food reward (rather than just push) with tool	↑	↑	Trap-tube	Bonobos, chimpanzees, gorillas, and orangutans	Mulcahy and Call, 2006
• Food retrievable with finger rather than tool		↑	Two-trap box	Chimpanzees	Seed et al., 2009
• Inedible test stimuli (tokens)	↑	↑	Quantity discrimination	Olive baboons and long-tailed macaques	Schmitt and Fischer, 2011
• Food type difference between test stimuli and rewards					
	=	=		Brown capuchins	Gazes et al., 2018
• Edible test stimuli	↑	↑		Brown capuchins	Addessi et al., 2008; Gazes et al., 2018
• High quality rewards	↑	↑		Brown capuchins and squirrel monkeys	Gazes et al., 2018
• Low probability of success by chance (large number of test stimuli)	(↑)	↑	Memory (modified after TTB)	Common marmosets, common squirrel monkeys	Schubiger et al., 2016
	(↑)	↑	Uncertainty monitoring (computerized metacognition task)	Rhesus monkeys and capuchin monkeys	Beran et al., 2014
				Capuchin monkeys	Beran et al., 2016
• Multimodal exploration of test stimuli	↑	↑	Visual discrimination	Capuchin monkeys	Carducci et al., 2018
*Social cognition tasks*					
• Competitive rather than cooperative experimenter cues	↑	↑	Object-choice	Chimpanzees	Hare and Tomasello, 2004
• Experimenter’s cue already in place when subject enters test area	(↑)	↑			Barth et al., 2005
				Common marmosets	Burkart and Heschl, 2006
• Low probability of random success	(↑)	↑			
• Eye contact at time of experimenter’s communicative cue	(↑)	↑		Bonobos, chimpanzees, and orangutans	Mulcahy and Call, 2009; Mulcahy and Suddendorf, 2011
• Larger distance between test stimuli	↑	↑			
	↑	↑	Perspective taking	Chimpanzees	Hare et al., 2000; Karin-D’Arcy and Povinelli, 2002
**b) Opportunistic testing**					
*Excluding subjects with motivational issues*					
• Excluding subjects who need more testing time	↑	=	Inhibitory control (A-not-B and detour-reaching, adapted from the TTB) and memory	Common marmosets and squirrel monkeys	Schubiger et al., 2019
			Quantity discrimination and reversal learning (adapted from the TTB)	Common marmosets	

*No effect: =, negative effect: ↓, positive effect: ↑. Note: Symbols in parentheses () indicate that participation/attention and response motivation were only indirectly assessed.*

In comparative psychology, the construct validity of cognitive tasks appears particularly important when assessing individual and species differences in one cognitive ability or domain (e.g., inhibitory control: MacLean et al., 2014). However, this is arguably not equally the case when individuals and species are tested with test batteries, i.e., sets of cognitive tasks that assess performance in various cognitive abilities that are not isolated from each other (but may overlap to some degree; Huber, 2017; Ramus, 2017), provided these tasks do measure aspects of cognition. Therefore, the central issue in comparative psychology is to establish internal task validity more generally, that is whether a task truly measures individual and species differences in the cognitive abilities it is supposed to measure rather than variation in factors that are not primarily of cognitive nature and might bias the outcomes of a study. Such biases may be especially problematic when the same individuals are tested with test batteries consisting of several tasks that might not be controlled for confounding factors. In test batteries that are used to evaluate general intelligence (*g*), the main question is whether individual performance is correlated across tasks, and such correlations can be the result of confounds rather than a true positive manifold (i.e., a positive correlation of unbiased individual cognitive performance scores; Burkart et al., 2017a). While some confounding factors are overt and obvious and can be relatively easily dealt with, such as sensory-motor differences, other factors may not be as straightforward.

### Overtly Necessary Preconditions for Valid Species Comparisons

Evolutionarily distantly related vertebrate taxa such as mammals and birds whose cognitive performance is often compared using the same tasks and test-setups, can vary greatly in sensory-physiological and morphological variables such as vision, olfaction and dexterity. Such differences are even more pronounced when comparing cognition of vertebrate taxa to invertebrate taxa such as cephalopods or bees. Essential for valid cognitive comparisons is that three basic preconditions are met. Every tested individual should possess (i) the sensory (e.g., visual or auditory) abilities to easily perceive the test apparatus and distinguish the test stimuli, (ii) the motor skills to easily handle the test apparatus and (iii) sufficient motivation to participate in and attend to the cognitive task at hand (Schubiger, 2019). The first two preconditions are related to test fairness (i.e., comparable conditions for all individuals to understand a cognitive task and perform well in it) and are arguably best met in conservative comparisons between evolutionary closely related taxa such as within primates or birds, whereas motivational and attentional aspects can probably not be equally well controlled by restricting comparisons to closely related taxa.

### Reducing Sensory-Motor Influences on Cognitive Performance by Testing Primate Species

Even when conducting cognitive species comparisons within the primate order, some differences in sensory-motor skills remain that might affect individual test performance if not considered when constructing test apparatuses. For instance, many strepsirrhines (lemurs and lorises) are nocturnal and possess limited color vision (particularly along the red-green spectrum) and dexterity, while they appear to rely more on olfaction than haplorrhines (monkeys and apes), most of whom are diurnal and have excellent stereoscopic color vision. Yet different haplorrhine primate species, and even different individuals within a single species, also vary to some degree in perception and dexterity (King, 2016; Heldstab et al., 2016). Importantly, such differences have to be considered when planning and conducting cognitive tests. For instance, nocturnal strepsirrhines such as mouse lemurs were tested under infrared light to adapt testing conditions to their activity period (Kittler et al., 2018; Fichtel et al., 2020). In marmosets, most males are dichromats (red-green color blind) whereas most females have trichromatic vision owing to a cone receptor polymorphism (Pessoa et al., 2005; Freitag and Almeida Pessoa, 2012). In order to ensure these individuals were equally able to perceive the test stimuli, researchers either used yellow and blue colored test stimuli or refrained from using colored stimuli altogether by using black and white ones instead (Schmon, 2011; Strasser and Burkart, 2012; Schubiger et al., 2015, 2016, 2019). Furthermore, one central characteristic of the callitrichids (marmosets and tamarins) is that they have claws rather than typical primate hands with fingernails, which needs to be taken into account when designing tasks in which subjects need to manipulate objects (as e.g., Schubiger et al., 2016; Schubiger et al., 2019).

While test designers and experimenters can largely control sensory-motor confounding factors by using appropriate test apparatuses, they may have limited control over several other non-cognitive factors during testing. For instance, motivational aspects remain a potential source of bias on cognitive performance. Examples are inner states and predispositions that affect how individuals approach and attend to their non-social (including cognitive tasks) and social environment (including the human experimenter and the cognitive test situation). However, deliberate test designs and analytical methods might alleviate some of these issues (e.g., Schubiger et al., 2015).

Starting from primate cognition studies, we here review recent studies that exemplary address (1) how individual differences in several non-cognitive factors affect participation and performance in cognitive tasks commonly used for within and between-species comparisons and (2) how aspects of test design and human-induced biases directly or indirectly affect cognitive performance. Finally, we evaluate (3) how recent studies that used cognitive test batteries may be affected by such effects.

## Overview of Studies on Non-Cognitive Factors That Potentially Confound Cognitive Performance

Individuals can differ considerably in terms of non-cognitive factors (i.e., intrinsic ones such as individual differences in personality, emotion and motivation) and different species differ in social factors (such as levels of social tolerance or social organization or structure) that might affect their cognitive task performance. Recent comparative cognition studies have started to assess a number of such internal (subject-related) and external (test design-related) non-cognitive factors and their potential effects on cognitive performance. Here, we review several relevant studies and their findings regarding whether they affected cognitive task performance or not (see also Table 1).

### Internal (Subject-Related) Factors

#### Intrinsic Factors

##### Personality-related motivational differences

###### Personality

At least some personality traits have been shown to affect cognitive performance in non-human primates. For instance, trait anxiety as assessed by the monkeys’ sustained reaction to a loud noise, was negatively correlated with the cognitive performance of long-tailed macaques in a reversal learning task (Toxopeus et al., 2005). Personality traits of non-human primates have also been assessed in a comparable manner to humans using the Hominoid Personality Questionnaire (HPQ; King and Figuereo, 1997; Weiss, 2017). Traits such as openness (behaviorally associated with the time an individual devotes to playing with conspecifics) and assertiveness (behaviorally associated with an individual’s aggressive behavior toward conspecifics) were associated with the participation and performance of capuchin monkeys *(Sapajus apella)* in two training tasks that preceded cognitive testing. Subjects with more open or less assertive personalities and particularly those exhibiting a combination of both trait expressions were more motivated to participate and also performed better in the training tasks than less open and highly assertive subjects (Morton et al., 2013).

###### Temperament and neophobia

In non-human primates, temperament or neophobia is generally measured as the latency to approach novel objects, humans, or food. Bolder chimpanzees *(Pan troglodytes*) and orang-utans *(Pongo pygmaeus)* approached such novel situations more quickly and performed better in some physical subtests of the PCTB than their shyer conspecifics. In the social subtests, however, individual differences in temperament were not associated with the apes’ cognitive performance in either domain (Herrmann et al., 2007). The opposite pattern was found for ruffed lemurs in that shyer subjects who took longer to approach and spent less time in the testing area performed better in the social PCTB tasks than their bolder conspecifics. This was not the case for ring-tailed lemurs whose temperament did not correlate with performance in the physical or social tasks of the PCTB (Fichtel et al., 2020). Olive baboons, particularly males, spent more time next to new objects than long-tailed macaques and showed a shorter approach latency toward new stimuli than long-tailed macaques, particularly females. Their performance in the PCTB, however, was not associated with these species and sex differences in temperament (Schmitt et al., 2012). Importantly thus, species can interact with influences of personality, temperament and neophobia in predicting cognitive outcomes.

###### Emotional reactivity

While an individual’s temperament and personality traits are fairly stable over time, its emotional reactivity may differ depending on the context and be particularly strongly expressed in the test situation. Common marmoset (*Callithrix jacchus*) subjects who showed a strong spontaneous emotional reaction to the experimenter and the test situation participated in fewer trials of an object permanence task than their less emotionally reactive conspecifics (Schubiger et al., 2015). Elevated emotional arousal, which the marmosets visibly and auditorily expressed (via piloerection of the tail as well as increased vigilance, mobbing vocalizations and avoidance behavior), was particularly apparent in the majority of male individuals and affected their attention in trials in which they participated. However, when strict pre-defined stop criteria were applied to abort a test session when a subject’s state of elevated emotional arousal persisted, their cognitive performance was not affected.

#### Social Factors

##### Rearing conditions, housing conditions, and previous contact with humans

Individual differences in rearing conditions, housing conditions and previous contact with humans may affect cognitive performance in primates, which is of particular importance when testing and comparing primates from more heterogenous populations such as in different zoos, sanctuaries and in the wild. One example is that a young individual who has abundant opportunity to learn socially from its adult conspecifics (ideally by being mother-raised), is able to acquire a larger set of cognitive skills than an individual who is deprived of this opportunity (such as an orphan growing up with peers; reviewed in van Schaik and Burkart, 2011).

Adult chimpanzees, for instance, who had experienced *impoverished rearing conditions* in the first two years of their lives, performed poorly in a reversal learning task (as reflected in their lower transfer indices, a measure of cognitive flexibility) than their mother-raised conspecifics (Davenport et al., 1973), even though all chimpanzees had been living at the same facility during the last six years prior to cognitive testing.

*Enriched rearing conditions*, on the other hand, favored whether chimpanzees (Gallup et al., 1971) and gorillas (Patterson and Cohn, 1994; Posada and Colell, 2007) showed evidence of mirror self-recognition. Moreover, infant chimpanzees growing up in enriched nursery-care conditions developed better socio-cognitive abilities than their conspecifics raised in standard nursery care, particularly those abilities related to joint attention (as measured by 30 tasks of the Bayley Scales of Infant Development for human infants: BSID, Bayley, 1969) and cooperation (as rated by the experimenter using the Infant Behavior Record: IBR, Bayley, 1969; Bard et al., 2014). A more recent study with zoo-housed chimpanzees, however, found that being *mother-reared vs. nursery-reared* was not associated with how well adult chimpanzees performed in the subset of 13 physical and social PCTB tasks they were tested with (Hopkins et al., 2014).

Being *housed with conspecifics* in zoos and sanctuaries and *being cared for by humans* allows individuals to be more curious and explorative toward their surroundings than their single-housed conspecifics. Such favorable rearing and housing conditions, most likely in combination with higher exposure to human artifacts, facilitated performance in the Orangutan Test Battery (OTB, Damerius et al., 2017b).

Individual differences in orangutans’ *previous contact with humans* have recently been quantified by a new composite measure that assesses individual differences in the subjects’ behavioral response to unfamiliar humans, the Human Orientation Index (HOI, Damerius et al., 2017a). Individuals who had been more exposed to humans exhibited higher HOI-scores than those with limited human exposure, were more explorative and also more successful than less human-oriented orangutans at solving the honey-box task of the OTB, in which they had to use tools to extract honey from a wooden apparatus (also see Damerius et al., 2019).

##### Social organization and social tolerance

Primate species differ regarding their social organization such as the spatiotemporal cohesion of the societies they live in (Kappeler and van Schaik, 2002). Fission-fusion societies, for instance, are characterized by dynamic group compositions with changing associations between individuals both in time and in strength (Dunbar, 1988). In haplorrhine primates more generally, ape and monkey species living in social systems characterized by a *high degree of fission-fusion dynamics*, such as great apes and spider monkeys, performed better in two physical cognition tasks similar to the ones of the PCTB (“spatial memory” and “shape”) and in several inhibitory control tasks from other sources (Amici et al., 2008, 2010).

Primate species also differ considerably in terms of *social tolerance levels*. Tolerant primate societies are characterized by less steep dominance hierarchies, low levels of conflicts without clear directionality, and feeding in close proximity (Jaeggi et al., 2010a; Fichtel et al., 2018). All these factors can facilitate highly social behavior such as cooperating in solving problems and prosocial acts such as proactively sharing food with conspecifics (Jaeggi and van Schaik, 2011; Burkart et al., 2014). For instance, in cooperative tasks (that required two individuals to simultaneously lift heavy stones to uncover food items or pull-in a feeding platform they would both be able to access) more tolerant macaque and ape species performed better than less tolerant ones. In particular, Tonkean macaques performed better than rhesus macaques (Petit et al., 1992) and bonobos better than chimpanzees (Hare et al., 2007). Likewise, socially more tolerant macaque species performed better than less socially tolerant macaque species in an inhibitory control task and in one PCTB task from the social scale (pointing cups) that tests the subject’s ability to communicate to the experimenter in which location food has earlier been placed by a second person, but not in any other PCTB tasks (Joly et al., 2017). Finally, the cooperative callitrichid monkeys (marmosets and tamarins with higher levels of *allomaternal care*) exhibit generally high levels of social tolerance, which appears to facilitate performance in socio-cognitive tasks such as social learning or cooperative problem solving compared to their less tolerant sister taxa (Burkart and van Schaik, 2010, 2016). Importantly, at least in primates, social tolerance can also differ considerably between different groups of the same species (Jaeggi et al., 2010b; Cronin et al., 2014; Burkart, 2015) and thus mask or exaggerate potential species differences in cognitive performance.

#### Demographic Factors

The potential effects of individual differences in demographic factors on cognitive performance are well known and often taken into account by comparing balanced, unbiased samples to avoid confounding effects or statistically controlled in studies using cognitive test batteries.

##### Social rank

An individual’s rank in its social group is typically inferred via the number of decided conflicts between individuals gathered via focal observations. An individual’s social rank did not affect its cognitive performance in any of the PTCB tasks in olive baboons, long-tailed macaques (Schmitt et al., 2012) or ring-tailed and ruffed lemurs (Fichtel et al., 2020).

##### Sex

Male chimpanzees in Herrmann et al.’s (2007) PCTB study performed better than females in the space scale and male children performed better than female children in the quantities scale. However, in Hopkins et al.’s (2014) chimpanzee study, a subject’s sex did not affect its performance in the PCTB and neither did it in baboons and long-tailed macaques, although male and female individuals differed in terms of temperament in these two Old World primate species (Schmitt et al., 2012). Similarly, sex did not influence the performance of three lemur species in the PCTB (Fichtel et al., 2020). In marmosets, males were generally more easily emotionally aroused in the test situation than females and less food motivated to participate in the cognitive tasks (Schubiger et al., 2015). In addition, males were often more attentive to their surroundings than the test apparatus in front of them, which is in line with males showing more vigilance behavior in the wild (Koenig, 1998). Importantly, however, because they were given the opportunity to leave the test situation as soon as they became unmotivated and inattentive, and because inattentive trials were excluded from the analysis, they performed comparably to their female conspecifics (Schubiger et al., 2015).

##### Age

Generally, as in humans, cognitive abilities recruiting fast and flexible (fluid) mental processes and maintaining information such as executive functions (e.g., inhibitory control) are also predicted to decrease with increasing age in non-human primates (e.g., Deary et al., 2009). Cognitive abilities that improve with experience, on the other hand, such as many social ones, are predicted to increase over an individual’s lifetime (for a review see Burkart et al., 2017a). Interestingly, the opposite pattern was found in chimpanzees (Lacreuse et al., 2014) in that age had a positive effect on individual performance in the physical cognition tasks of the PCTB (with the exception of the spatial memory task) but a negative effect on their performance in two of the socio-cognitive tasks (attentional state, gaze following). Similarly, older chimpanzees and bonobos performed better in some of the physical tasks (causality scale) but not as well as younger individuals in some social tasks (Theory of Mind scale, Herrmann et al., 2010). In the other studies applying the PCTB, no such age-effects on cognitive performance were reported (Schmitt and Fischer, 2011; Fichtel et al., 2020). Another interesting finding was that high levels of curiosity appeared to alleviate cognitive decline in marmosets (Gokcekus, 2020). While marmosets’ ability to inhibit directly reaching into a transparent barrier (detour-reaching tasks) declined with age in individuals with low curiosity scores, this ability remained stable in particularly curious individuals and in some cases even increased with age. Hence, the influence of age on performance in cognitive tests is not straightforward.

### External (Test Design-Related) Effects on Performance

#### Task Format

Task format, i.e., the way in which a cognitive task is designed and how (many) test stimuli are presented to the subject, not only generally affected cognitive performance in several empirical comparative cognition studies but it sometimes did so in different ways in different species.

##### Physical cognition tasks

One physical task that has been used to test the causal understanding of non-human primates is the *trap-tube task*, originally designed by Visalberghi and Limongelli (1994) testing capuchin monkeys. In the original trap-tube task, a food reward was placed in a transparent tube that contained a trap in its middle. In order to retrieve the reward, the subject had to use a stick tool and push the reward out of the tube while preventing it from falling into the trap. To ensure that chimpanzees were not using simple distance rules rather than causal reasoning, the trap was later moved to the side and the reward placed in the tube’s middle instead (Limongelli et al., 1995), but this could not rule out other simple rules such as always pushing the food away from the trap, which chimpanzees tended to do even when the trap was inverted and non-functional (Povinelli, 2000). The strongest evidence for great apes’ causal understanding of the trap-tube problem comes from studies using modified task versions. For instance, great apes performed better in a modified trap-tube task, in which the tube was wider so that the apes could also use the stick tool to *rake-in the reward* (thereby pulling it away from the trap) rather than having to push the reward away from the trap (Mulcahy and Call, 2006). This suggests that improving the ecological validity of the task, which allowed the apes to use the tool in a more natural way, helped reveal their causal understanding. In a further modified version, the *Two Trap Box task*, the reward was placed on a shelf that had a trap on each end (one of which was blocked depending on the trial). Through the transparent front of the box, the subject had visual access to the test apparatus and could use its finger to move the reward away from the trap *without requiring a tool*, which made the task easier for chimpanzees (Seed et al., 2009). A similar task version consisting of a box with six channels each containing a trap was used in the OTB (Damerius et al., 2019), and a considerable number of individuals succeeded, suggesting that the causal problem was easier to solve when they could use their fingers instead of a tool.

In a *quantity discrimination task* (requiring subjects to choose the larger of two amounts of items), two Old World monkey species, olive baboons (*Papio anubis*) and long-tailed macaques (*Macaca fascicularis*) performed better when the test stimuli were *inedible* (i.e., tokens) as opposed to edible items (i.e., raisins). Interestingly, the same monkeys performed equally well with edible test stimuli when the food type of the edible items they were rewarded with differed from the *food type* of the test stimuli (Schmitt and Fischer, 2011). Contrarily, capuchins monkeys (*Cebus sapajus apella*) performed better with *edible* stimuli than tokens (Addessi et al., 2008; independent of food types: Gazes et al., 2018) and generally better than squirrel monkeys (*Saimiri sciureus*). When the quality of food rewards was modulated, both New World monkey species performed better with *higher quality rewards* independent of how long they had to wait to be rewarded (Gazes et al., 2018). This heterogeneous influence most likely emerged because highly attractive rewards on the one hand increase an individual’s motivation, but on the other hand elicit prepotent reactions and thus can increase demands on inhibitory control. Depending on which influence prevails, high quality rewards can both increase and decrease task performance.

Task format can also include how stimuli are presented, e.g., only visually or visually and haptically. This turned out to influence performance in a *visual discrimination task*, in capuchin monkeys (*Sapajus* spp.). They were more successful at distinguishing between two objects when they had access to *haptic* in addition to purely visual information by being allowed to touch and manipulate the objects suggesting they benefited from this multimodal exploration (Carducci et al., 2018).

Finally, task formats can vary with regard to *chance probabilities of success*. Modifications of these probabilities in physical cognition tasks revealed that common marmosets and common squirrel monkeys performed better in a *memory task* in which they had a choice between nine containers, only one of which was baited with a food item, than in the original two-choice version of the task. Lowering the chance probability of success from 50 to 11% made wrong choices in the nine-choice memory task more costly and is likely to have indirectly enhanced the monkeys’ motivation to favor an appropriate rather than a random choice strategy and thus more reliably assessed their memory decline over increasingly longer time delays (Schubiger et al., 2016). Similarly, capuchin monkeys showed better evidence of uncertainty monitoring in a computerized *metacognition task* by more often selecting the escape option when chance levels of success were lower than when they were higher, whereas rhesus macaques appeared less sensitive to higher costs of incorrect choices (Beran et al., 2014, 2016).

##### Social cognition tasks

One of the most extensively used social cognition test paradigms in comparative psychology is the *object choice task* (originally developed by Anderson et al., 1995) in which the experimenter sits or stands opposite the subject, hides a food item in one of at least two containers, and then provides the subject with at least one (visual and/or auditory) communicative cue to indicate the food’s location before the subject is allowed to choose one of the containers. Primates, especially great apes, have been shown to perform poorly in comparison to many other animal species, including distantly related mammals such as canids as well as birds (e.g., Bräuer et al., 2006; Giret et al., 2009). However, although this has been interpreted as the apes’ inability to understand human-given communicative cues, the original test setup used for primates differed from the one used for other animal species and several modifications to the primate version substantially improved the apes’ performance (for a detailed review see Mulcahy and Hedge, 2012), the most relevant of which we list here.

###### Competitive experimenter cues

In one object-choice study, chimpanzees performed better in an object-choice task if the experimenter’s cue was competitive in that he extended his arm in an attempt to grab the baited container rather than pointing at it in a cooperative manner (Hare and Tomasello, 2004). However, as Mulcahy and Hedge (2012) pointed out, the competitive task version also differed from the cooperative one in that the former included a potential inhibition component and higher costs of an incorrect choice. Before being able to choose one of the two containers, the apes had to open a corresponding transparent panel in the testing window. Having to do so might not only have prevented them from making ambiguous choices but also enhanced their motivation to attend to the experimenter’s cue(s). This in turn might have helped them to perform better in the competitive task version.

###### Experimenter’s cue already in place when subject enters the test area

In Barth et al.’s (2005) object choice study, chimpanzees performed poorly in the original version of the task in which the experimenter provided the communicative cue (head and eyes directed toward the baited container) only once the subject was directly in front of the experimenter. In contrast, the same chimpanzees located the reward much more successfully in a modified task version, in which the experimenter initialized the gaze cue before the subject entered the test area. This indicates that when entering the test area, the chimpanzees immediately looked into the direction of the experimenter’s cue and as they were approaching, they veered in this direction which would result in them arriving at and choosing the correct container. Similarly, in a marmoset study, the subjects’ access to choosing containers was restricted until the experimenter had provided the pointing gesture toward the baited container which might have facilitated basic inhibitory and attentional processes required to make correct choices (Burkart and Heschl, 2006).

###### Lowering the chance probability of random success

Burkart and Heschl’s (2006) task version also differed from other object-choice studies in that marmosets were presented with nine containers, only one of which contained a reward. This version was directly compared to a two container version, and the marmosets performed much better in the first one, which probably enhanced their motivation to attend to the experimenter’s cues because incorrect choices involved a higher cost than in the traditional object choice task with two choice options. Together with similar findings on chance probabilities from two physical cognition tasks (i.e., memory and metacognition) mentioned earlier, this suggests that lowering the chance probability of making correct choices at random by increasing the number of available choice options may positively affect performance in tasks across cognitive domains.

###### Increasing the distance between test stimuli

Other modifications to the original object choice task, such as increasing the distance between the containers in which the food item is hidden, have also been shown to positively affect the performance of bonobos, chimpanzees, and orangutans in object choice tasks (e.g., Mulcahy and Call, 2009). The distance between test stimuli also turned out to be responsible for discrepant results in *perspective-taking tasks* that assess whether the subject knows if a conspecific individual present in the test situation can see a reward (i.e., because it is visible from both the subject’s and the conspecific’s point of view) or not (i.e., because a barrier obstructs the conspecific’s view). Initial findings by Hare et al. (2000) suggesting that chimpanzees knew what their conspecific could see could not be readily replicated (Karin-D’Arcy and Povinelli, 2002), but it turned out that this was owing to variation in spatial factors of the set-up. In the meantime, this paradigm has been applied to a variety of species with varying results, and it is not entirely clear which differences represent true species differences and which ones may be affected by spatial factors too (e.g., capuchin monkeys: Hare et al., 2003 vs. common marmosets: Burkart and Heschl, 2007).

###### Establishing eye contact when giving the cue

Although it is effortful to establish eye contact with some non-human primate subjects and this is not possible with all species (e.g., owing to gaze aversion or being perceived as a threatening gesture), ensuring in this way that the subject is attentive to the experimenter’s cues has been shown to improve the performance of bonobos, chimpanzees, and orangutans in the object choice task (Mulcahy and Call, 2009; Mulcahy and Suddendorf, 2011).

#### Opportunistic Testing

##### Excluding Subjects With Motivational Issues

One specific issue of comparative psychology is that not all species and not all individuals within a given species are equally motivated to continually participate in cognitive tasks. An individual’s lack of motivation to do so can critically affect the course of a study because the individual will require substantially more than the allocated or available testing time to complete the cognitive tasks. This can be particularly problematic in cognitive studies with non-human primates because access to respective testing facilities is often temporally limited, which constrains the time available for a study. Researchers often deal with this constraint by following an opportunistic approach of only testing individuals who readily participate and are most likely to complete the tasks in the available testing time and excluding those who are not. However, such opportunistic testing might bias a study’s outcome if the excluded individuals not only differ from the selected ones in motivational factors but also in terms of cognitive abilities.

In a recent study with common marmosets and common squirrel monkeys, this issue has been addressed by including less consistently motivated individuals and allowing them additional time to complete the six tasks of a cognitive test battery at their own pace (Schubiger et al., 2019). A direct comparison of individuals who needed additional *testing time* to those who were consistently motivated showed that both groups performed equally well in all tasks. This suggests that opportunistic testing and the selection bias that results from it does not necessarily affect a study’s outcome. Whether this also applies to other species still needs to be established.

## How Are Individual and Species Comparisons Using Cognitive Test Batteries Affected by Non-Cognitive Factors?

When using test batteries to assess individual differences within a species, priority is given to the same individuals completing all tasks. This is because obtaining a complete data set enables researchers to conduct factor-analytical performance analysis whereas dropouts would complicate this approach. A second goal is to obtain large enough sample sizes to reach sufficient statistical power. As current data suggest, using an opportunistic (as opposed to a randomized) approach by only selecting those individuals as subjects who are most likely to stay consistently motivated and complete all tasks is not a major issue. At least not as long as researchers report that and why this approach was followed and as long as dropouts and their performance in the few tasks they completed are also reported in detail.

However, some individuals (or species) might also require more time to get used to a new task because they are neophobic and more cautious when approaching the test apparatus for the first time. It is therefore advisable to allow every subject to familiarize itself with the basic test apparatus and to only start testing when the subject appears comfortable with all components. Highly neophilic individuals, on the other hand, tend to approach and get used to the test apparatus much more quickly with the risk that some of these individuals might also more quickly lose interest once the task is not novel anymore. In order to enable later replications of a study, it is therefore important to describe in detail how subjects were familiarized with the tasks prior to testing, how their motivation was regained if necessary, and which criteria were used to objectively decide when a test session started and when it had to be aborted.

Besides opportunistic testing, using several experimenters rather than just one is another way to test as many subjects as possible with all tasks in a limited testing period. Although training different experimenters to use the same standardized methods helps reducing experimenter effects that might bias the subjects’ cognitive performance, a certain risk of such unintended biases remains. Herrmann et al. (2007), for instance, used five different experimenters in the original PCTB study, including two experimenters with the rule that every subject was tested by the same experimenter with all tasks. Since one group of chimpanzees performed better than the other, Herrmann and her colleagues could not tease apart in how far these differences were purely cognitive in nature or also experimenter-induced.

Another issue is that not all subjects are equally motivated to participate in food-reward tasks and different food types do not have the same value for all individuals. One way of limiting such individual differences is to use tokens as test stimuli rather than food items (e.g., Addessi et al., 2008; Schmitt and Fischer, 2011). However, subjects need to be trained to use tokens, which limits the usefulness of this approach, particularly for large-scale species comparisons because test battery tasks should not require any previous experience. Quantity discrimination tasks in which the subject has to choose the larger of two quantities (of food or token items) to pass a trial have shown to be particularly susceptible to the type of test stimuli and rewards (Schmitt and Fischer, 2011; Gazes et al., 2018). Regardless of whether subjects chooses between two amounts of tokens or food items, the number of rewards usually corresponds to the chosen amount of test stimuli. However, this procedure differs from the one used in all other test battery tasks in which the subject usually only receives one reward in case of a correct choice, which could be one possible explanation why the task appeared to be difficult for squirrel monkeys in Gazes et al.’s (2018) study. Therefore, in the quantity discrimination task of their adapted test battery, Schubiger et al. (2019) used two amounts of edible “tokens” of low food quality as test stimuli, which made them interesting enough to attend to the task (and more interesting than non-food tokens) but not desirable as rewards. If the subject correctly chose the larger amount, it was, as in all other tasks, rewarded with one highly desirable food item. They found that the dropout rate in the quantity discrimination task was particularly low in comparison to most other tasks and the marmosets performed better in this task than in most others. Whether this was a consequence of the setup and reward contingency remains to be determined in future studies.

The most comprehensive test battery currently available for non-human primates is the PCTB consisting of a physical and social cognition scale that each comprise several subtests amounting to a total of 16 cognitive tasks (Herrmann et al., 2007). Initially, the PCTB was applied to the largest sample of great apes (chimpanzees and orangutans) that had ever been tested in comparative psychology and to 2.5 years-old human children who outperformed both ape species in most social but not in the physical cognition tasks. In the last decade, the full PCTB or parts of it (ranging from six to 13 tasks) have been used to assess and compare the cognitive abilities of ten other non-human primate species (with some minor adaptations). Besides an independent chimpanzee sample (tested with 13 tasks: Hopkins et al., 2014) the tested species included bonobos *(Pan paniscus*, Herrmann et al., 2010) one small ape species (lar gibbons, *Hylobathes lar*, *Yocom, 2010*, tested with six tasks), five Old World monkey species (olive baboons, *Papio anubis*, Schmitt et al., 2012; longtailed-macaques, *Macaca fascicularis*, Schmitt et al., 2012; Joly et al., 2017; Barbary macaques, *Macaca sylvanus*, rhesus macaques, *Macaca mulatta*, and Tonkean macaques, *Macaca tonkeana*, Joly et al., 2017) and three lemur species (tested with all tasks; black-and-white ruffed lemurs, *Varecia variegata*, ring-tailed lemurs, *Lemur catta*, and mouse lemurs, *Microcebus murinus*, Fichtel et al., 2020). In addition, four bird species (parrots) have recently also been tested with the full PCTB (African grey parrots, *Psittacus erithacus*, blue-headed macaws, *Primolius couloni*, blue-throated macaws, *Ara glaucogularis*, and great green macaws, *Ara ambiguous*, Krasheninnikova et al., 2019). Unanticipatedly and in contrast to previous meta-analytic studies (Deaner et al., 2006; Reader et al., 2011), the primate studies found that overall Old World monkeys and lemurs (who as strepsirrhines represent the evolutionarily most distant primate relatives of great apes) performed largely comparable to great apes, particularly in the social scale. Contrarily, all four parrot species performed inferior to great apes in both the physical and social scale of the PCTB (Krasheninnikova et al., 2019). This was unanticipated because parrots (besides corvids and owls) belong to the birds with the largest brain size and parts of their brains have been described as homologous to the mammalian neocortex (Jarvis et al., 2005; Güntürkün and Bugnyar, 2016). Based on their powerful brains and their remarkable cognitive abilities that have been demonstrated in several tasks and sometimes been considered to match or even exceed those of non-human primates (e.g., Pepperberg, 2006), the parrots were expected to perform relatively well in the PCTB.

Three explanations appear most plausible for this arguably unexpected pattern of results: (i) the tested species do not differ in terms of cognitive abilities (which would be in line with Macphail’s null hypothesis), (ii) small differences in task designs rather than cognitive ability masked species differences in cognitive performance, or (iii) the levels of task sensitivity were not appropriate to identify between-species variation and instead led to ceiling (i.e., mainly very high performance scores) or floor effects (i.e., mainly very low performance scores). While the first possibility appears unlikely to explain the primate and parrot findings based on what is known about their cognitive abilities, the two other two possibilities or a combination of the two (depending on the tasks and species) appear more plausible.

The lack of clear-cut performance differences between the different primate taxa points to ceiling effects in most tasks of the PCTB with relatively good performance levels in all species most likely owing to the relatively low task sensitivity (Fichtel et al., 2020). Moreover, all PCTB studies with monkeys and lemurs also found floor effects for at least one physical (tool use) and one social cognition task (social learning). In fact, only great apes passed the tool use task that required the ability to use a stick tool to rake a food reward into reach whereas no other primate species did. Doing so might have been too challenging for species exhibiting either a medium (baboons, macaques) or low (lemurs) level of precision grip (Torigoe, 1985; Kittler et al., 2018). This is not surprising because, even in captivity, great apes use stick tools more often and more skillfully than other primate species.

Comparably to the primates, all tested macaw species also performed relatively poorly in the tool-use task despite other parrot species (such as Goffin’s cockatoos, *Cacatua goffiniana*) having been shown to be skillful at using a stick to retrieve food in previous experiments. According to Krasheninnikova et al. (2019), this indicates that morphological rather than cognitive constraints such as their longer maxilla and a less muscular tongue made it difficult for the macaws to maneuver the stick and pull the food reward into reach.

The social learning task of the PCTB, for which another floor effect was found, required subjects to solve a problem using the same solution that a human experimenter had demonstrated, i.e., retrieving a food item out of a transparent or opaque tube using the same behavioral actions. It is not surprising that human children performed better than great apes in this task because children have been shown to over-imitate actions of adults by even copying unnecessary or unsuccessful steps or methods of a human demonstrator whereas chimpanzees did not (Horner and Whiten, 2005). In addition, however, children could learn from a conspecific demonstrator whereas all other species had to learn from a hetero-specific demonstrator, the human experimenter. Among non-human primates, great apes possess the most similar preconditions to children in that their hands and manipulation skills resemble those of humans the most (Heldstab et al., 2016). Consequently, a social learning task in which subjects could learn from a conspecific demonstrator and that is adapted to the manipulative skills of monkeys and lemurs might have been more informative (Fichtel et al., 2020).

A striking result was, however, that the parrots performed at chance-levels in most of the tasks of the PCTB, indicating that non-cognitive factors as well as aspects of task design may have played a role. Particularly in the space scale that largely consisted of object permanence tasks, all primates outperformed the macaws despite parrots having been shown to pass such invisible displacement tasks in previous studies, and even before reaching adulthood. As Krasheninnikova et al. (2019) suggested, having to choose containers by touching them with their beaks might have made it more demanding for the parrots to inhibit prepotent impulses to touch containers. Based on earlier findings on parrots’ numerical cognitive abilities, the African grey parrots and macaws would also have been expected to perform much better in the quantity tasks. The authors’ finding that many individuals seemed to choose in a random manner in many PCTB tasks, particularly in those that involved only two choice options, is in line with earlier findings on primates that subjects may not always be motivated to attend to the task and use an appropriate choice strategy when they have a 50% probability of making a correct choice by chance and being rewarded (e.g., Burkart and Heschl, 2007; Schubiger et al., 2016, 2019; Fichtel et al., 2020).

It is important to mention that while great apes only received one to three trials per task of the PCTB, other primates (Old World monkeys and lemurs) and parrots received up to six trials per subtest. In the object-permanence tasks of the physical scale, for instance, the monkeys and lemurs received six trials so that all spatial positions and combinations of the baited cups were evenly distributed. In principal, participating in the double amount of trials might have given them an advantage over the great apes in that they had the opportunity to learn and perform better across trials. However, the monkey and lemur results were stable and there were no learning effects from the first to the second half of trials, which makes it unlikely that they had a substantial learning advantage over the great apes (Schmitt and Fischer, 2011; Fichtel et al., 2020).

Therefore, the most plausible explanation for the apparent lack of cognitive differences between various primate species based on individual performance scores in the PCTB, is that some of the tasks are not valid or sensitive enough to reveal differences between species (Fichtel et al., 2020). For instance, all primate species from lemurs to great apes performed very well in the spatial memory task of the PCTB’s space subscale, which was basically an object permanence task for most species as it involved keeping track of two food items placed in two of three cups without a delay between the baiting of the cup and the subject’s choice. Only the transposition task, in which keeping track of the food item became more demanding, revealed some species differences, which indicates that this task’s level of difficulty was appropriate to distinguish between species while the other tasks of the space subscale led to ceiling effects.

The floor effects in the primates’ and parrots’ performance demonstrate that researchers are facing a trade-off when constructing cognitive test batteries. While the test set-ups need to be sufficiently abstract to also identify a putative domain-general cognitive ability rather than only capturing narrow domain-specific adaptations, the task apparatuses also need to be ecologically valid enough to be easily perceived and handled by all individuals. While closely related species can be largely tested with the same test apparatuses and setups with only minor adjustments, large-scale species comparisons might require more changes. This might particularly be the case for species with low dexterity levels or those who have to use their beaks (birds) or noses (e.g., canids and elephants) to handle the test apparatus and make responses. As the parrot results suggest, validated tasks should be used that can be adjusted for as many species as possible while keeping the cognitive task itself as similar as possible. This is an immense challenge for comparative psychology that yet needs to be accomplished.

One task that may be promising for meaningful species comparisons is the reversal learning task (Rumbaugh, 1971) in which all individuals (of every species) first have to master an initial discrimination by reaching the same predefined criterion. In the actual test, the discrimination is then reversed and it is assessed how quickly the subjects switch to the new discrimination in relation to their pre-reversal performance (Transfer Index). Despite their timeliness, reversal learning tasks have rarely been part of cognitive test batteries. Moreover, the pivotal measure, the Transfer index, has only been determined in one study that used a modified version of the TTB task to test marmosets (Schubiger et al., 2019). The latter task version was optimized in that two differently patterned black-and-white plates were used as test stimuli under which the reward could be hidden, rather than presenting and hiding the reward in the experimenter’s hands (two different colors of gloves, including a green one) as in the original TTB. This optimization was applied in order to minimize potential effects of individual differences in the subjects’ color-vision as well as potential experimenter effects. Including reversal learning tasks in future test batteries will allow to compare the cognitive flexibility of different individuals and species regardless of how long they needed to reach the initial criterion. Doing so would also help to establish whether new experimental data support findings from metanalytical research in which reversal learning performance was the best predictor of general intelligence across species (Deaner et al., 2006).

Most importantly, however, our findings illustrate the importance of conducting basic validations of cognitive tasks and test batteries in comparative psychology before applying them to a broader range of different species. This can be achieved by establishing that the cognitive tasks truly measure differences and similarities in aspects of cognition rather than other aspects that are not primarily cognitive in nature. One possible way of doing so is to first establish that each cognitive task (of a future test battery) identifies intra- and inter-individual cognitive variation in one species by assessing which potentially confounding factors can be ruled out or have to be controlled as far as possible. In a second step this species could then be compared to its closest evolutionary relatives and then to more distant ones. Another way is to use one or a small number of established and validated cognitive tasks to compare the cognitive abilities of a wide range of different species (e.g., as in the Many Primates project, ManyPrimates et al., 2019a, b). Importantly, in the latter type of studies, relatively overt differences in sensory-motor aptitudes need to be considered, particularly when comparing evolutionarily distantly related taxa (e.g., such as primate to bird species). While minor adaptations to the basic test setup and apparatus might be sufficient in some tasks, other tasks might require more substantial modifications to be suitable for a wide range of species. This is a challenge, which might demand (repeated) re-designing of tasks that turn out to be unsuitable but a challenge worth pursuing in order to establish that cognitive tests truly capture cognitive differences and similarities between individuals and species. Optimized test batteries consisting of tasks that are largely controlled for these factors will more accurately measure if and how species compare or differ in terms of cognitive abilities without non-cognitive factors playing a substantial role. This will provide a more solid base for meaningful inferences and conclusions regarding how these similarities or differences may have evolved.

## Conclusion

In this review, we provided an overview of recent studies that assessed (subject- and test design-related) non-cognitive factors that may confound the outcomes of primate cognition tasks in general and primate test batteries in particular. In order to take into account sensory-motor species-differences, we have largely focused on studies with primates rather than other mammals or birds (with the exception of the PCTB that was applied to several parrot species). Our findings from these typical comparative cognition tasks suggest that individual differences in non-cognitive internal (subject-related) factors (such as personality-mediated intrinsic motivational factors) affected cognitive performance primarily via attention, which in principle can be controlled or at least quantified. Depending on the individuals and species tested, differences in social and demographic factors may positively or negatively affect performance. Unless cognitive comparisons specifically account for the influence of such factors on cognitive performance, it is therefore essential to report these potential sources of variation and control them if possible.

We conclude that non-cognitive factors are a minor issue if experimenters ensure they only test attentive individuals who are motivated to use appropriate response strategies. This is best achieved by either presenting more than two choice options to the subjects whenever possible or by using modified two-choice task versions that prevent motivational issues. Since relatively small differences in task format and test procedures can have major effects on the outcomes of comparative cognition studies, it is essential to report the testing procedure and individual results in detail, ideally supplemented with video clips. While basic aspects of internal task validity can thus be improved by establishing that the tasks measure at least cognition *per se*, the more specific issue of construct validity remains. Since all cognitive abilities represent constructs and as such have to be inferred from cognitive test performance, each construct needs to be carefully defined before its validity can be established. Although establishing construct validity in a top-down (theory-based) rather than a bottom-up approach (statistically) would be ideal, doing so is extremely challenging (but see Burkart et al., 2017a, b for recommendations on how to achieve this).

In sum, test design remains the major issue of contemporary comparative psychology and it is essential that researchers validate and redesign cognitive tests, if needed, in order to ascertain that the tasks accurately capture cognitive abilities. Once the sensitivity, reliability, and internal validity of cognitive tasks have been established, these tasks can then be integrated into test batteries and applied to an increasingly wide range of species. This will also help establishing their external validity, i.e., if they measure the same cognitive abilities in different species. Such evaluated test batteries that only include tasks with established internal and external validity will then hopefully provide a solid base for future cognitive comparisons and further our understanding of the evolution of mind, including the human mind.

## Author Contributions

MS, CF, and JB wrote the review together. All authors contributed to the article and approved the submitted version.

## Conflict of Interest

The authors declare that the research was conducted in the absence of any commercial or financial relationships that could be construed as a potential conflict of interest.
